# Biophysical Regulations of Epigenetic State and Notch Signaling in Neural Development Using Microgroove Substrates

**DOI:** 10.1021/acsami.2c01996

**Published:** 2022-07-13

**Authors:** Chia-Chen Hsu, Andrea Serio, Sahana Gopal, Amy Gelmi, Ciro Chiappini, Ravi A. Desai, Molly M. Stevens

**Affiliations:** † Department of Materials, Imperial College London, Exhibition Road, London SW7 2AZ, U.K.; ‡ Department of Bioengineering, Imperial College London, Exhibition Road, London SW7 2AZ, U.K.; § Institute of Biomedical Engineering, Imperial College London, Exhibition Road, London SW7 2AZ, U.K.

**Keywords:** neural tissue engineering, neural stem cell, neuron, topography, micropatterning, epigenetics, Notch signaling

## Abstract

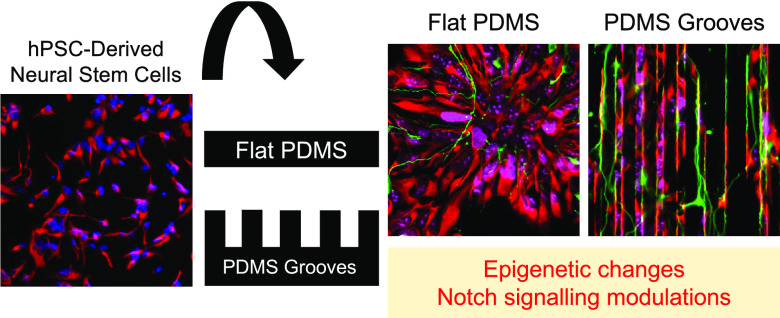

A number of studies have recently shown how surface topography can alter the behavior and differentiation patterns of different types of stem cells. Although the exact mechanisms and molecular pathways involved remain unclear, a consistent portion of the literature points to epigenetic changes induced by nuclear remodeling. In this study, we investigate the behavior of clinically relevant neural populations derived from human pluripotent stem cells when cultured on polydimethylsiloxane microgrooves (3 and 10 μm depth grooves) to investigate what mechanisms are responsible for their differentiation capacity and functional behavior. Our results show that microgrooves enhance cell alignment, modify nuclear geometry, and significantly increase cellular stiffness, which we were able to measure at high resolution with a combination of light and electron microscopy, scanning ion conductance microscopy (SICM), and atomic force microscopy (AFM) coupled with quantitative image analysis. The microgrooves promoted significant changes in the epigenetic landscape, as revealed by the expression of key histone modification markers. The main behavioral change of neural stem cells on microgrooves was an increase of neuronal differentiation under basal conditions on the microgrooves. Through measurements of cleaved Notch1 levels, we found that microgrooves downregulate Notch signaling. We in fact propose that microgroove topography affects the differentiation potential of neural stem cells by indirectly altering Notch signaling through geometric segregation and that this mechanism in parallel with topography-dependent epigenetic modulations acts in concert to enhance stem cell neuronal differentiation.

## 1 Introduction

The topography of the extracellular matrix (ECM) with various spatial arrangements and structural features influences a wide range of cellular responses, including attachment, migration, proliferation, differentiation, and functionality.^1−4^ By application of advanced micro- and nanofabrication techniques, bioengineered substrates can now be fabricated to recapitulate topographical characteristics of the native ECM.^3,5^ Surface-based microgrooves and nanogrooves have been shown to enhance neuronal differentiation and guide neurite extension in different cell types, including immortalized cell lines (e.g., Luhmes cells),^6^ human embryonic stem cells (hESCs),^7^ human induced pluripotent stem cells (hiPSCs),^8^ and human neural stem cells (hNSCs).^9^ For example, nanogrooves could induce hESC neuronal differentiation without induction of soluble factors.^7^ Additionally, poly(dimethylsiloxane) (PDMS) microgrooved substrates with features of 1 μm in width and in depth significantly enhanced human mesenchymal stem cell (MSC) proliferation, neuronal differentiation, and cytosolic calcium responses with application of potassium chloride (KCl) compared to flat surfaces and microgrooves with wider features of 2 and 4 μm in width.^10^ Although an abundance of studies have shown how topography regulates cell behaviors in the nervous system, the underlying mechanisms have not yet been fully elucidated.

Epigenetics (e.g., DNA methylation, histone modification, and chromatin remodeling) refers to gene expression changes without alteration of a DNA sequence.^11^ Because epigenetics plays a critical role in development and in response to external stimuli, a few studies have investigated the effects of topography on cellular epigenetic regulation. Human MSCs cultured on microgrooved substrates exhibited an increase in histone acetylation and a decrease in histone deacetylase (HDAC) activity.^12^ Microgrooved topography has shown to improve fibroblast reprogramming efficiency into iPSCs by modulating the cells’ epigenetic status, wherein the microgrooved surfaces downregulated HDAC activity and upregulated WD repeat-containing protein 5 (WDR5) and a subunit of H3 methyltransferase, leading to increased acetylation and methylation of histone H3.^13^ Previously, hESCs cultured on nanogratings showed that as neuronal differentiation progressed, the H3K9me3 expression level increased and became better organized on day 7 on nanogratings.^14^ However, the mechanisms underpinning topographical regulation of the cell epigenetic state using microgrooves in neural systems have not been elucidated. Previously, a few epigenetic markers have been reported for their regulatory roles during neural development. When pluripotent genes and non-neural lineage genes acquire H3K9me3, long-term repression of these genes occurs during differentiation of ESCs into NSCs.^15^ Repression of numerous nonneuronal and neuronal progenitor genes is also related to H3K9me3 modifications in adult neurons.^15^ Activation of neuronal genes through histone acetylation has also implicated an important role during neuronal differentiation. After treatment with an HDAC inhibitor, valproic acid (VPA), the proliferation of adult neural progenitors was decreased and neuronal differentiation was increased without increasing gliogenesis.^10^ Via chromatin immunoprecipitation (ChIP) analysis, it was found that VPA-induced neurogenesis was accompanied by the association of AcH4 with the promoter of Ngn1 (a proneural transcription factor).^16^

Besides epigenetic mechanisms, the Notch signaling pathway is essential in controlling neural cell proliferation and differentiation in embryonic and adult brains.^17^ Notch signaling is activated when the transmembrane ligands (i.e., delta or serrate (jagged)) of one cell interacts with the heterodimeric Notch receptor of an adjacent cell. The binding triggers sequential proteolytic activation, wherein presenilin−γ-secretase complex cleavage releases the intracellular domain of the Notch receptor (NICD); the NICD translocates to the nucleus^18,19^ and activates and recruits the DNA-binding effector suppressor of hairless (Su(H); CBF1/RBPjκ) and the nuclear protein mastermind (MAM), further triggering Notch target gene transcription.^20,21^ It is known that a key Notch signaling event is the upregulation of basic helix–loop–helix (bHLH) transcriptional repressors,^22^ which repress proneural gene expression and therefore inhibit neuronal differentiation.^17^ Notch signaling is involved in maintenance of NSCs,^23^ glia–neuron cell fate decisions,^24^ and regulation of terminally differentiated neuron behavior.^25,26^ Because Notch signaling highly depends on cell-to-cell contacts, controlling the spatial geometric arrangement modulating cell–cell interactions could potentially regulate Notch signaling and the downstream cellular responses.

In this study, we utilized a microgrooved PDMS platform with 3 and 10 μm depth grooves (noted as 3 μm grooves and 10 μm grooves, respectively) both with 10 μm ridge/groove width, compared to a topographically featureless (flat) control to examine how topography affects neuronal populations derived from human pluripotent stem cells (hPSCs), including hESCs and hiPSCs, focusing on the effects in cell alignment, stiffness, proliferation, and neural differentiation. The 10 μm ridge/groove width was designed for single cell confinement in the grooves in accordance with the soma diameter of the NSCs (usually ∼10 μm)^27^ to examine the effects resulting specifically from cell spatial constraint. With the use of an *in vitro* modeling system based on clinically relevant and human cells, we obviated the discordance between human and animal/immortalized cell lines, which have been widely used previously. We particularly focused on how topography modifies the epigenetic landscape and how it also modulates Notch signaling. To the best of our knowledge, it was unknown prior to our study how epigenetic and Notch signaling axes were impacted by microgrooved topographical cues presented to neural systems. We propose that the topography-induced alterations to the epigenetic landscape and Notch signaling act in concert to enhance stem cell neuronal differentiation.

## 2 Materials and Methods

### 2.1 Cell Culture

The hESC H9 line (WiCell, USA) and the human episomal iPSC line (Thermo Fisher Scientific, UK) were maintained on Matrigel (Corning Inc., UK) coated culture plates by using chemically defined mTeSR1 medium (Stem Cell Technologies, UK) and Essential 8 media (Thermo Fisher Scientific, UK), respectively. hESCs and hiPSCs colonies were passaged when they reached 80–90% confluency by dissociation with 1 mg/mL collagenase IV (Sigma-Aldrich, UK) and 0.5 mM EDTA (pH 8.0; Thermo Fisher Scientific, UK) in sterile PBS.

Cells were differentiated based on a previously published protocol with some modifications.^28^ When high confluency was reached, the hPSCs were differentiated into neuroectoderm via dual-SMAD signaling inhibition^29^ using a neural induction medium composed of Advanced DMEM/F-12 medium (Thermo Fisher Scientific, UK), 0.2% (v/v) B27 Supplement (Thermo Fisher Scientific, UK), 1% (v/v) N-2 supplement (Thermo Fisher Scientific, UK), 1% (v/v) GlutaMAX (Thermo Fisher Scientific, UK), and 1% (v/v) penicillin/streptomycin (Thermo Fisher Scientific, UK) supplemented with 10 μM SB431542 (Merck Millipore, UK), 2 μM InSolution AMPK inhibitor, Compound C (Merck Millipore, UK), and 1 mM *N*-acetylcysteine (Sigma-Aldrich, UK) for 6–7 days. NSCs were then passaged by using enzymatic dissociation and plated on laminin (Sigma-Aldrich, UK)-coated plates in NSCR base medium, composed of Advanced DMEM/F-12 medium (Thermo Fisher Scientific, UK), 0.2% (v/v) B27 Supplement (Thermo Fisher Scientific, UK), 1% (v/v) N-2 supplement (Thermo Fisher Scientific, UK), 1% (v/v) GlutaMAX (Thermo Fisher Scientific, UK), and 1% (v/v) penicillin/streptomycin (Thermo Fisher Scientific, UK). After 3–5 days, when hPSC-derived NSCs proliferated and formed neural rosette structures, the NSCs were maintained in F20 Medium, composed of NSCR Base Medium supplemented with 20 ng/mL FGF2 (PeproTech, UK). NSCs were passaged every 5–7 days on laminin-coated plates for the first 3–5 passages and on Matrigel-coated plates for later passages.

The hESC-derived NSCs were used in immunofluorescent experiments for densitometry, proliferation, and differentiation measurements while the hiPSC-derived NSCs were used for measurement of live NSCs’ mechanical stiffness using atomic force microscopy (AFM) and scanning ion conductance microscopy (SICM), focused ion beam-scanning electron microscopy (FIB-SEM) tomography analysis, and human cleaved Notch1 enzyme-linked immunosorbent assay (ELISA) experiments. Previously, studies have shown that hiPSCs and hESCs present the same neuronal differentiation potential and use the same transcriptional networks to generate NSCs and their neuronal lineages over the same developmental time course.^30,31^ Because of their proven similarities in the previous studies^30,31^ and in our study using immunoblotting (Figure S1), the results of hESC- and hiPSC-derived NSCs were presented and described as hPSC-derived NSCs in this study.

### 2.2 Fabrication of Microgrooved Substrates

Silicon wafers patterned with 10 μm ridge/groove width and 3 or 10 μm depth parallel microgrooves were fabricated by using standard soft-lithography techniques.^32^ Briefly, a layer of SU8-2002 photoresist was spin-coated onto a silicon wafer. The photoresist was then exposed to UV light through the patterned photomask, and the unpolymerized photoresist was subsequently rinsed away. PDMS substrates were fabricated based on a previously published protocol with some modifications.^12^ To create PDMS membranes with flat, 3 μm depth, and 10 μm depth parallel microgrooves, a SYLGARD 184 kit (Dow Corning) was used. Silicone elastomer was first mixed with the curing agent in a 10:1 weight ratio and vacuum degassed. The mixture was then spin-coated onto silicon wafers with microgrooved patterns for 1 min at 300 rpm to the thickness of ∼250 μm and cured at 120 °C for at least 30 min. The PDMS membranes were then removed from the template and cured at 60 °C for another 2 days and washed with 100% (v/v) ethanol before use. Fabrication was assessed with SEM (Figure S2).

### 2.3 Cell Seeding on Microgrooved Substrates

To change the hydrophobicity of PDMS and facilitate coating, micropatterned PDMS membranes were treated with oxygen plasma at a pressure of 0.5 mbar and a power of 35 W for 1 min by using Plasma Prep 5 (GaLa Instrumente, Germany). The PDMS membranes were immediately coated with 0.1 mg/mL PDL (Sigma-Aldrich, UK) under UV-treatment at room temperature for 15 min followed by three washes using molecular biology grade water and then coated with 10 μg/mL Laminin (Sigma-Aldrich, UK) at 37 °C for 2 h. hPSC-derived NSCs were detached with Accutase (Stemcell Technologies, UK), pelleted at 300*g* for 5 min, and resuspended in NSCR Neuron medium, composed of NSCR Base Medium supplemented with 10 ng/mL BDNF (R&D Systems, UK) and 10 ng/mL GDNF (R&D Systems, UK). 6.25 × 10^4^ cells were plated onto laminin-coated PDMS membranes in wells of a 48-well plate for fluorescent immunostaining assays, and 2 × 10^6^ cells were plated onto membranes in 6 cm dishes for protein collection for ELISA.

### 2.4 Immunofluorescent Assays for Densitometry, Proliferation, and Differentiation Measurements

hPSC-derived NSCs cultured on PDMS substrates were fixed with 4% (v/v) paraformaldehyde for 15 min, followed by permeabilization with 0.2% (v/v) Triton X-100 (Sigma-Aldrich, UK) for 10 min, and then blocked in 3% (v/v) goat serum (Sigma-Aldrich, UK) for 30 min at room temperature. Primary antibodies, including histone H3 (acetyl K9 + K14) (1:1000; Cell Signaling Technology, UK), histone H4 (acetyl K5 + K8 + K12 + K16) (1:1000; Abcam, UK), histone H3 (trimethyl K9) (1:1000; Abcam, UK), Ki67 (1:1000; Abcam, UK), Nestin, clone 10C2 (1:500; Merck Millipore, UK), and βIII-Tubulin, clone SDL.3D10 (1:1000; Sigma-Aldrich, UK) were incubated for 1 h, washed three times with PBS, and then followed by secondary antibodies (Alexa Fluor dyes; Life Technologies, UK) and 4′,6-diamidino-2-phenylindole (DAPI; Sigma-Aldrich, UK) for 30 min. The samples were mounted on glass slides by using FluorSave Reagent (Merck Millipore, UK) and stored at 4 °C in the dark. Images were acquired by using the EVOS FL Cell Imaging System (Life Technologies, UK) or a SP5MP/FLIM inverted confocal microscope (Leica, Germany).

Epigenetic modulations of cells were examined by using densitometric analysis with ImageJ 64 (ver. 2; National Institutes of Health, USA (NIH)). For densitometry, the mean fluorescence intensity (MFI) of the epigenetic markers was normalized to MFI of DPAI per nuclei. The data were represented as fold induction with respect to the flat PDMS control.

Nuclear circularity was calculated by using the following formula with ImageJ 64 (ver. 2; NIH):
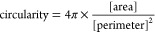
11.0 indicates a perfect circle, and values approaching 0.0 indicate increasingly elongated shapes.

NSC proliferation and differentiation were performed by using the “Cell Counter” plugin, ImageJ 64 (ver. 2; NIH). A proliferation marker, Ki67, and a neuronal marker, βIII-tubulin, were used as indications for cell proliferation and neuronal differentiation, respectively. The percentages of the proliferating cell population and the neuronal population were calculated by using the following formulas:
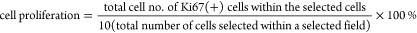
2
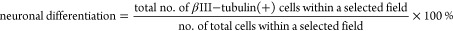
3

### 2.5 Live NSCs’ Mechanical Stiffness Measured by Scanning Ion Conductance Microscopy (SICM)

hPSC-derived NSCs (9.5 × 10^4^ cells/cm^2^) were first plated onto the PDMS substrates. After 48 h cell seeding, the stiffness of the live NSCs was measured by using scanning ion conductance microscopy (SICM). Pipettes with inner diameters of ∼100 nm were pulled from borosilicate glass (O.D. 1 mm, I.D. 0.5 mm, Intracel, Cambridge, UK) with a P-2000 laser puller (Sutter Instruments, USA). Ion current measurements were examined by using Axopatch 200B amplifiers (Molecular Devices, UK). Pipettes were filled with PBS, and a bias potential (200 mV) was used for imaging for each experiment. Traces were analyzed by using pClamp 10 (Molecular Devices, USA).

Samples were mounted in a 35 mm dish, and the medium was changed to warm L-15 during the experiment. Stiffness maps were generated by simultaneously recording three topographical images at progressively increased set points by using an approach speed of 30 nm/ms as previously reported,^33^ where the standard topographical measurement (*z* height) was acquired at the set point of 0.3%–0.4% of ion current drop compared to the reference (maximum) current at every image point.^34^ At this set point, only minimal stress in the range 0.1–10 Pa was exerted by the nanopipet on the cell membrane. The nanopipet was then further lowered to two consecutive set points (0.6% and 3%), and the corresponding heights were stored as separate image points before the pipet moved to the next horizontal location. Eventually, each image had recorded differential height maps representing sample strain as the pipet deformed the sample at two higher compressive stresses.

### 2.6 Young’s Modulus of the PDMS Substrate and Live NSCs Measured by Atomic Force Microscopy (AFM)

Flat PDMS substrates were facbricated according to section 2.2. hPSC-derived NSCs (9.5 × 10^4^ cells/cm^2^) were plated onto a 6 cm dish. Young’s moduli of the PDMS substrates and the live cells were measured and analyzed by using an AFM 5500 microscope (Agilent, USA). The measurements were performed by using an HQ:CSC38 tipless cantilever (MikroMasch, USA) with a spring constant of 0.04 N/m, modified with a silica sphere (*D* = 20 μm). The force measurements of the PDMS substrates were performed in ambient conditions and were performed across 20 × 20 μm^2^ areas per sample, two samples in total, with 32 force curves in each area while the force measurements of the live cells were performed in culture media and were performed by performing a force–indentation line scan across a cell with the cantilever placed directly over individual NSCs. Young’s modulus was then quantified by using the force–indentation curves identified, where the force versus distance curves were converted to force versus indentation curves and further fitted by the Hertz model for a spherical indenter by using the free software PUNIAS (http://punias.free.fr/).
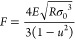
4*E* represents Young’s modulus, *R* the radius of the spherical indenter (10 μm), *u* the Poisson’s ratio, σ_0_ the indentation of the sample, and *F* the applied force. The fit was applied to a range of 2.5 μm indentation for the measurements of the PDMS substrates while the fit was applied to a range of 1 μm indentation for the measurements of the live cells to minimize the influence of the underlying PDMS substrate.

### 2.7 Focused Ion Beam-Scanning Electron Microscopy (FIB-SEM) Tomography

hPSC-derived NSCs were fixed with 4% (v/v) paraformaldehyde for 15 min and rinsed in 0.1 M sodium cacodylate buffer (Electron Microscopy Sciences, USA). The samples were then treated with 1% (v/v) osmium tetroxide (Electron Microscopy Sciences, USA) in 0.1 M sodium cacodylate buffer for 1 h, followed by two 5 min washes in double-distilled water. The samples were then incubated with 1% (w/v) tannic acid (Electron Microscopy Sciences, USA) in water for 1 h, followed by 1% (w/v) uranyl acetate (Electron Microscopy Sciences, USA) in water for a minimum of 2.5 h at room temperature. Finally, the samples were dehydrated in 20% (v/v), 30% (v/v), 50% (v/v), 70% (v/v), 80% (v/v), 90% (v/v), and 100% (v/v) ethanol for 5 min twice with four additional washes with 100% (v/v) ethanol for 5 min and embedded in resins (Epoxy Embedding Medium kit, Sigma-Aldrich). The resin embedded cell samples were mounted on stubs, sputter-coated with chromium (10 nm), and imaged with an Auriga cross beam focused ion beam-scanning electron microscopy (FIB-SEM; Zeiss, Germany). For FIB-SEM, the stage was tilted to 54°, and the samples were milled by using a current between 600 pA and 1 nA, yielding a slice thickness of 30 nm, and were imaged at a slice interval of 3 by a backscattering detector at an acceleration voltage of 1.6 keV. Images were segmented by using Amira (FEI, USA) and then reconstructed and analyzed by using Fiji and Volocity software (PerkinElmer, USA).

### 2.8 Human Cleaved Notch1 Enzyme-Linked Immunosorbent Assay (ELISA)

PathScan Cleaved Notch1 (Val1744) Sandwich ELISA Kit (Cell Signaling Technology, UK) was used according to manufacturer instructions. Briefly, 100 μL of samples was diluted with the sample diluent and added to the appropriate well. The microwell plate was sealed firmly and incubated at 4 °C overnight, followed by washes with the wash buffer. 100 μL of reconstituted detection antibody and 100 μL of reconstituted HRP-linked secondary antibody were then added to each well and incubated at 37 °C for 1 h and at 37 °C for 30 min, respectively, followed by washes with the wash buffer after each incubation. 100 μL of TMB substrate was added to each well and incubated at 37 °C for 10 min. Finally, 100 μL of STOP solution was added, and the absorbance values were measured at 450 nm with a SpectraMax M5 (Molecular Devices, USA). The acquired data were normalized and compared to the flat PDMS control in each experiment.

### 2.9 Statistical Analysis

For statistical analysis, all experiments were conducted at least three times throughout the study. Statistical tests and numbers of biological and technical replicates (*N* and *n*, respectively) are detailed in the figure captions. The one-way ANOVA with post hoc Tukey’s test was used throughout the study unless specified otherwise. A *p*-value of <0.05 was considered statistically significant, and all results represent mean ± s.e.m. unless specified otherwise. In the figures, ∗ represents *p* < 0.05, ∗∗ represents *p* ≤ 0.01, and ∗∗∗ represents *p* ≤ 0.001.

## 3 Results

### 3.1 Cells Align and Differentiate on Microgrooved Substrates

**Figure 1:**
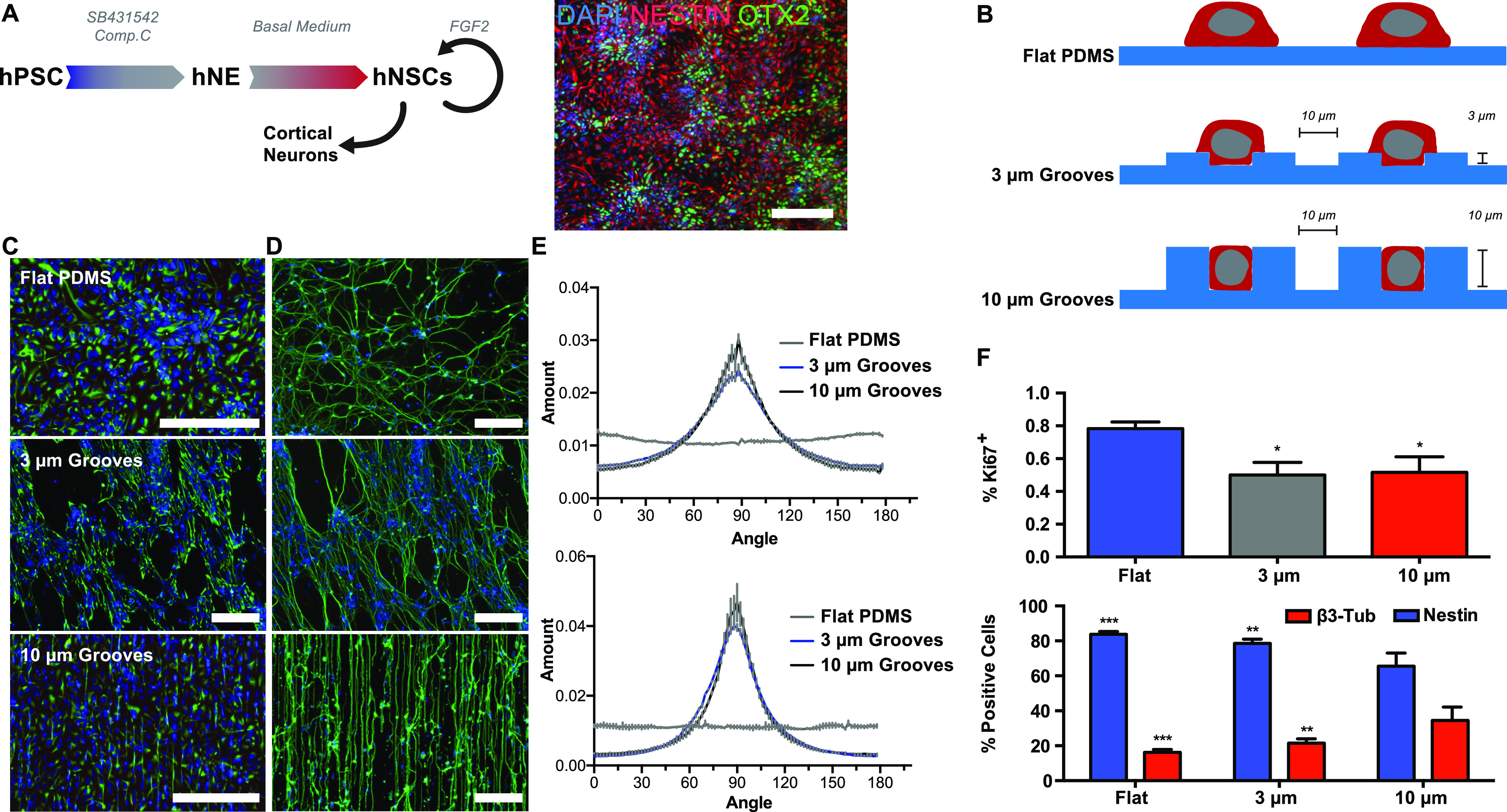
Cell alignment and differentiation of human neural stem cells (hNSCs) on microgrooved substrates. (A) Schematic of the neuronal differentiation process, where human pluripotent stem cells (hPSCs) are differentiated into hNSCs (hNE, human neuroepithelial cells; scale bar: 200 μm). (B) Schematic demonstrating cell attachment on different microgrooved tissue culture substrates, including flat PDMS (top), 3 μm grooves (middle), and 10 μm grooves (bottom). (C, D) Cell alignment of (C) NSCs (Nestin, green; DAPI, blue; scale bars: 200 μm) and (D) neurons (βIII-tubulin, green; DAPI, blue; scale bars: 200 μm) cultured for 14 days on flat PDMS, 3 μm grooves, and 10 μm grooves. (E) Quantification of cell alignment in three technical replicates (wells) from three different PDMS device plating using immunofluorescence image analysis of Nestin (top) and βIII-tubulin (bottom). (F) Cell proliferation (Ki67^+^; top) and neuronal differentiation (bottom) of hNSCs on day 14 (for the top panel, one-way ANOVA with post hoc Dunnett’s test was used; ∗ represents *p* < 0.05 compared to flat PDMS; *n* = 6; for the bottom panel, two-way ANOVA with post hoc Tukey’s test was used; ∗∗ represents *p* ≤ 0.01 compared to 10 μm grooves; ∗∗∗ represents *p* ≤ 0.001 compared to 10 μm grooves; *n* = 4).

To establish an experimental platform to study how topography affects neuronal populations derived from human pluripotent stem cells (hPSCs), we first confirmed neural differentiation of hPSCs following a modified version of a published protocol^28^ (see section 2.1) by immunofluorescent staining for Nestin and OTX2 (Figure 1A). We fabricated PDMS substrates presenting no topography (flat substrate), moderate topography (10 μm wide, 3 μm deep grooves), or high topography (10 μm wide, 10 μm deep microgrooves) (see section 2.2; Figure 1B). Previously, substrate stiffness and topography have been shown to synergistically influence cell behaviors, where substrates with lower stiffness permit cells to deform the surrounding substrates, thus dynamically modulating cellular mechanosensing.^35,36^ Similar to the range that has been previously reported,^37,38^ the Young’s modulus of the fabricated PDMS substrates is 2.9 ± 0.4 MPa measured by AFM (Figure S3), which is above the range of cell sensing regime (∼0–40 kPa),^39^ demonstrating that the substrates used in this study are rigid enough to decouple the observed effects from the influences exerted by cell-mediated substrate deformation. Coating the substrates with PDL and laminin allowed us to culture human neural stem cells (hNSCs) on substrates presenting varying topography (see section 2.3), induce differentiation on the substrates, and measure cellular alignment. Both hNSCs and differentiated neurons aligned on substrates presenting both moderate and high topography but not on control (flat) substrates (Figure 1C–E). We further assessed the efficacy of our differentiation protocol by measuring cell proliferation via Ki67 immunostaining and key neuronal stem cell and differentiation markers, Nestin and βIII-tubulin (see section 2.4; Figure 1F). It was found that there was a downregulation of Ki67 and Nestin as well as an upregulation of βIII-tubulin on the microgrooved platforms. The different trends of Nestin and βIII-tubulin expression are due to the fact that the two markers generally target mutually exclusive cell types, where the expression of Nestin is high in NSCs or neural progenitor cells while the expression of βIII-tubulin is high in postmitotic neurons and low or absent in NSCs or neural progenitor cells.^40,41^ Together, these data demonstrate that our microfabricated substrates can be used to simultaneously probe neuronal differentiation of hNSCs and substrate topography in one experimental platform. We therefore leverage this developed platform for the remainder of the study.

### 3.2 Microgroove Topography Impacts hNSC Nuclear Shape

**Figure 2:**
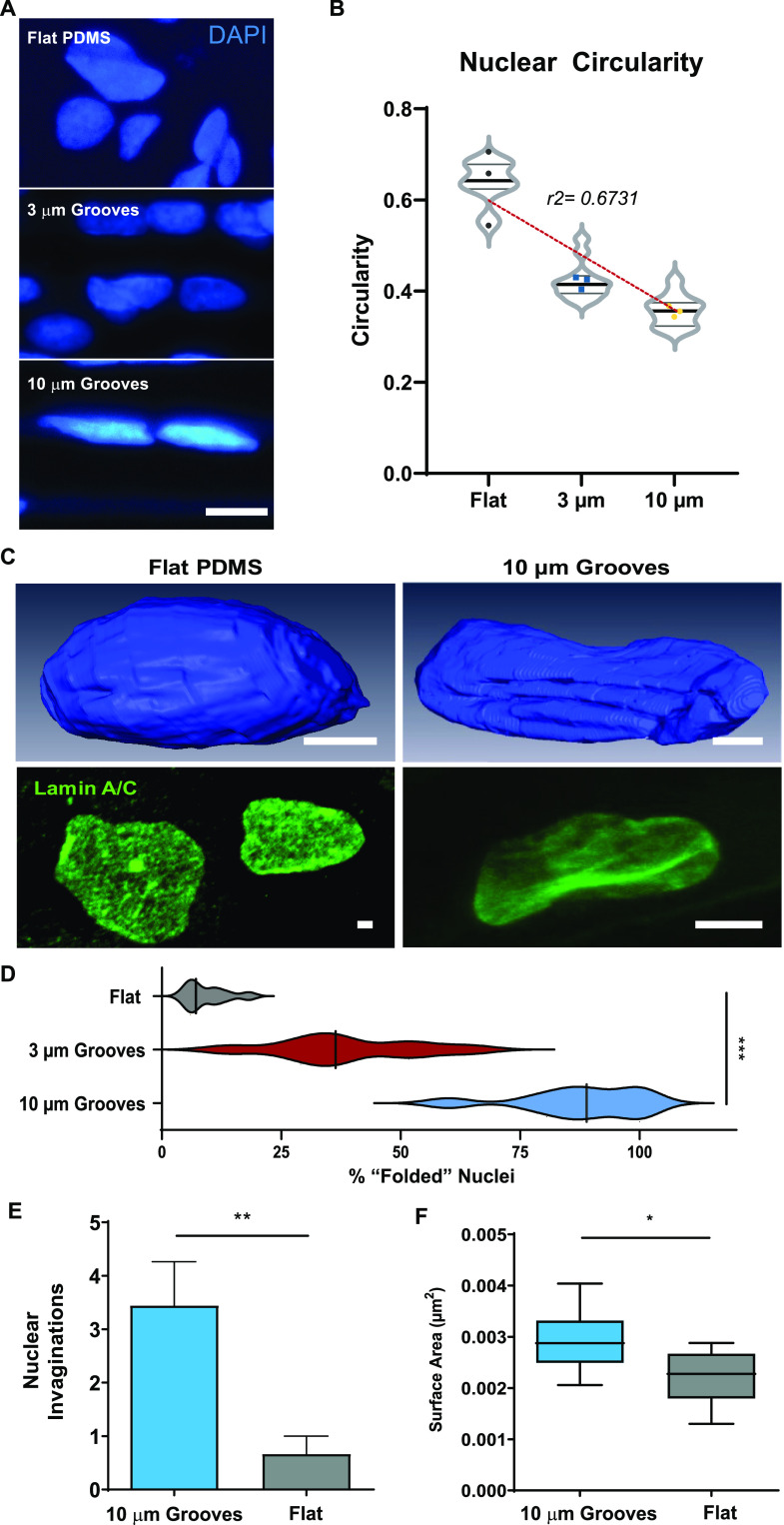
Effects of microgrooved topography on nuclear morphology in hNSCs on day 2. (A) Nuclear staining (DAPI, blue) and (B) nuclear circularity of hNSCs on different microgrooved substrates. (C) Nuclear morphology of cells on the flat PDMS and the 10 μm grooves (top: 3D reconstruction of FIB-SEM imaging of cell nuclei; bottom: confocal imaging of nuclear envelope Lamin A/C; scale bars = 2 μm). Quantification of nuclear morphology by (D) percentage of folded nuclei, (E) number of nuclear invaginations, and (F) nuclear surface area of cells on the 10 μm grooves and flat PDMS (“Flat”) (Mann–Whitney U-test was used; ∗ represents *p* < 0.05; ∗∗ represents *p* ≤ 0.01; ∗∗∗ represents *p* ≤ 0.001; *N* = 3, *n* = 9; lines in the violin and the box–whiskers plots display the following values: lower border/line, first quartile; middle line, median; upper border/line, third quartile; lower whisker, minimum; upper whisker, maximum).

Microgrooves have shown to provide control over cell alignment and uniaxial mechanical strain, which could further exert changes in nuclear shape and nuclear volume.^12,13,32,42^ We observed hNSCs to align on microgroove substrates (Figure 1C–E) and hypothesized that microgroove topography might similarly impinge also on hNSC nuclear shape. To test this hypothesis, we seeded hNSCs and cultured them on substrates presenting microgrooves, stained for nuclei and quantified nuclear shape by calculating nuclear circularity (see section 2.4). A circularity of 1.0 indicates a perfectly circular nuclear shape, and a circularity value approaching 0.0 indicates an increasingly elongated nuclear shape. Cells on microgroove substrates but not flat controls had elongated nuclei (Figure 2A). Nuclei of hNSCs on microgroove substrates presenting no, moderate, and high topographic cues had monotonically decreasing circularity values, indicating that nuclear shape is impacted by microgroove topography (Figure 2B).

Detailed three-dimensional changes to nuclear geometry were not revealed by our measurements of nuclear circularity. To examine nuclear shape at ultrahigh resolution and in 3D, we used focused ion beam scanning electron microscopy (FIB-SEM) tomography to study cells on flat and the deepest microgrooved substrate, 10 μm depth grooves, which results in more significant topographical effects on cell behaviors compared to the 3 μm depth grooves. FIB-SEM imaging revealed that cells which were docked within the 10 μm depth grooves (Figure S4) not only appeared more elongated as suggested by circularity data but also had more invaginations than their control counterparts on flat substrates (Figure 2C, top panels). Confocal imaging of Lamin A/C provided biomolecular details and supported FIB-SEM data showing altered nuclear morphology, by showing that substrates presenting microgroove topography impacted fine-grained nuclear shape including nuclear folding (Figure 2C, bottom panels; Figure 2D). To measure nuclear invaginations observed in the FIB-SEM images, we analyzed the images and found that substrates presenting microgrooves had significantly more nuclear invaginations relative to their control counterparts (Figure 2E). The orientation of the nuclear invaginations seemed more aligned with nuclear polarization on the 10 μm depth grooves compared to the 3 μm depth grooves and the flat substrate (Figure S5). Further analysis of FIB-SEM images revealed that nuclei on substrates presenting microgrooves had significantly higher surface area relative to their control counterparts (Figure 2F), consistent with more intricate three-dimensional nuclear features we initially observed in the FIB-SEM and Lamin A/C images. Collectively, our data indicate that microgroove topography impacts nuclear shape and suggest that concomitant biophysical cellular changes may also be present.

### 3.3 Microgroove Topography Impacts hNSC Stiffness

**Figure 3:**
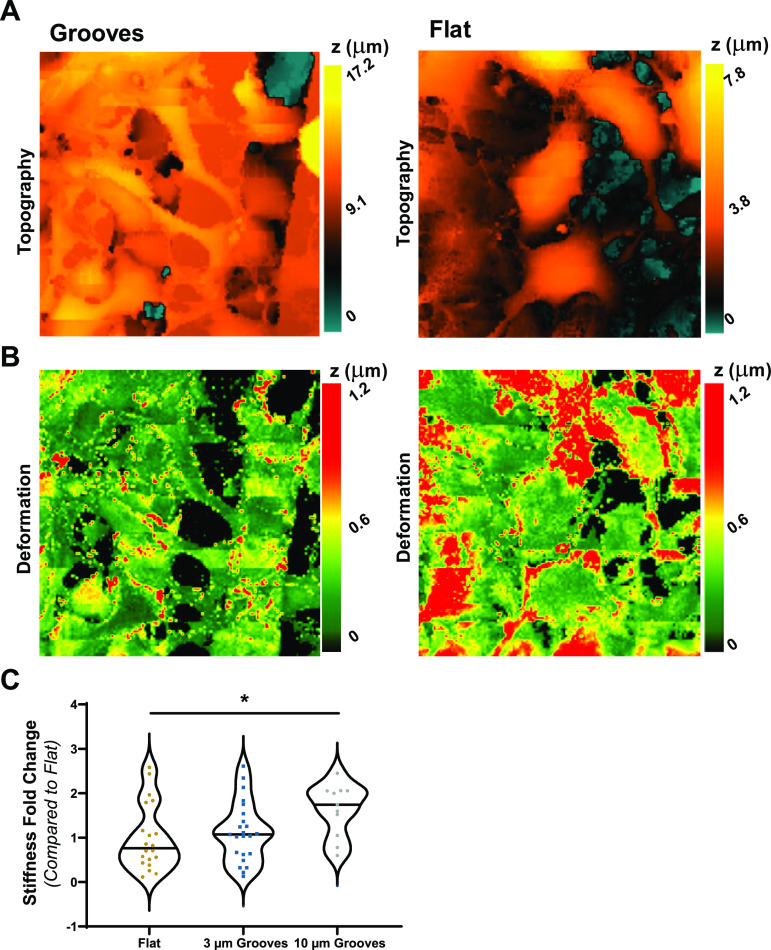
Stiffness of cells on microgrooved substrates. (A) Topography and (B) deformation maps of cells on the 10 μm grooves (Grooves) and the flat PDMS (Flat) measured by SICM with a scan area of 50 μm × 50 μm. (C) Fold change of cell stiffness on different microgrooved substrates measured by AFM (one-way ANOVA on ranks with post hoc Dunnett’s test was used; ∗ represents *p* < 0.05 compared to flat PDMS; lines in the violin plots display median; *n* = 20 for Flat, 23 for 3 μm Grooves, and 11 for 10 μm Grooves).

Our data clearly indicate that hNSCs both align and have altered nuclear morphology on substrates presenting microgrooves. While it is well-known from other studies cells can respond to environmental cues, such as rigidity and topography of the underlying substrate, by regulating their cell shape, internal cytoskeletal tension, and stiffness,^43−45^ it remains unclear whether hNSCs similarly change biophysical parameters including topography, deformability, and stiffness on substrates presenting microgrooves. To test this, we cultured hNSCs on substrates presenting microgrooves, differentiated them, and took measurements of topography and deformation via scanning ion conductance microscopy (SICM) and stiffness via atomic force microscopy (AFM). SICM indicated cellular topography and deformation of the cells in response to applied force (Figure 3A,B), and AFM measurements revealed that cell stiffness on the 10 μm grooves was significantly higher than corresponding cells on flat substrates (Figure 3C). It is known that cells can actively monitor cell shape and substrate rigidity and modulate their focal adhesion, cytoskeletal structure, and contractile force to dynamically alter their own stiffness.^35^ Collectively, our data showing cell shape changes, nuclear shape changes, and cellular stiffness changes indicate a corresponding biochemical and biophysical change in hNSCs on substrates presenting microgrooves.

### 3.4 Microgroove Topography Modulates Epigenetic Markers

**Figure 4:**
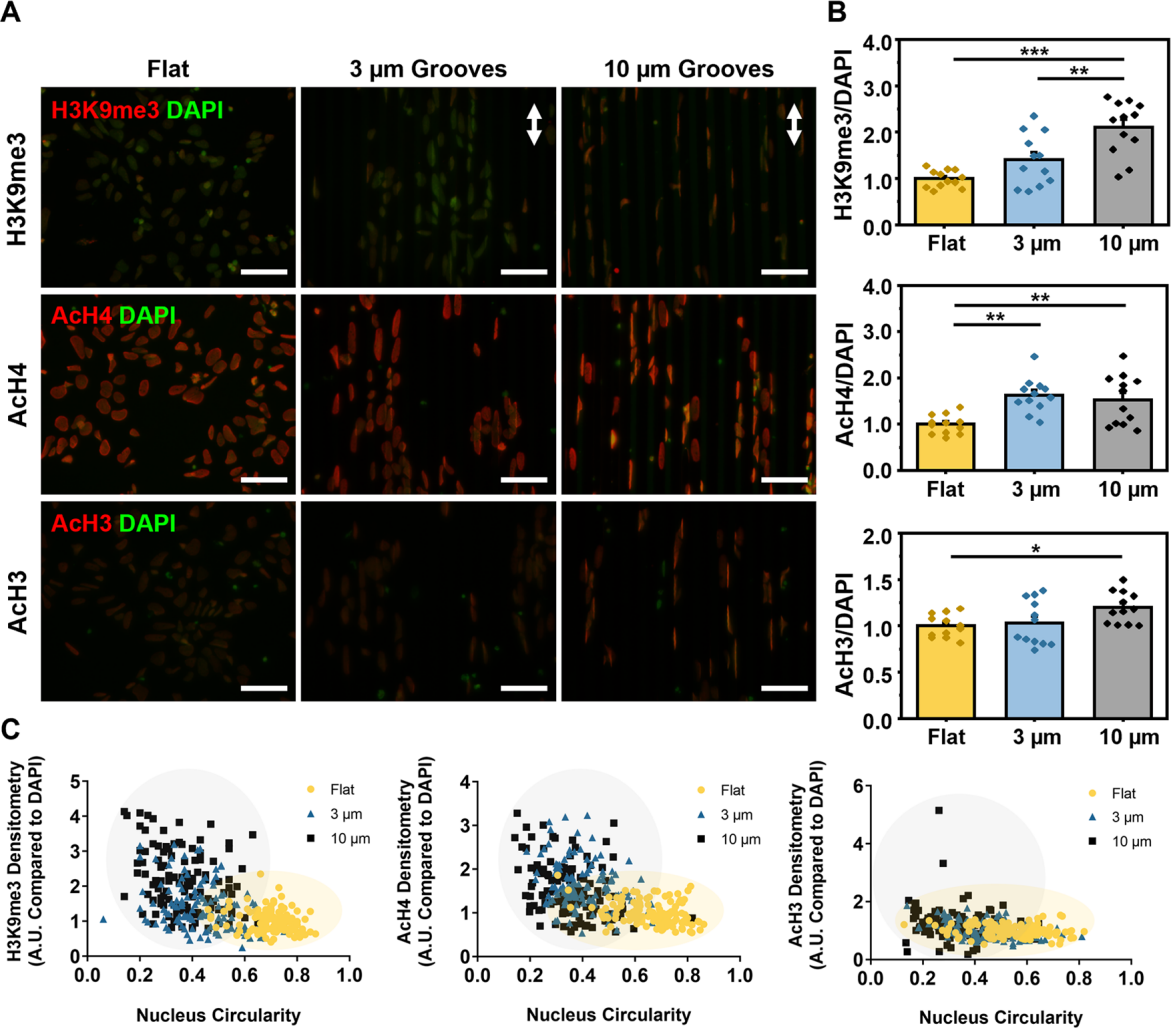
Microtopography modulated epigenetic status of hNSCs on day 2. (A) hNSCs on different PDMS substrates were stained with epigenetic markers, including H3K9me3, AcH4, AcH3 (red), and DAPI (green) on day 2 (white arrows: direction of grooves; scale bars = 50 μm). (B) Epigenetic changes of H3K9me3 (top), AcH4 (middle), and AcH3 (bottom) were analyzed by densitometric analysis (the fluorescence intensity of histone modifications is normalized to DAPI and then normalized to the flat PDMS) (one-way ANOVA with post hoc Tukey’s test was used; the results represent means ± s.e.m. ∗ represents *p* < 0.05; ∗∗ represents *p* ≤ 0.01; ∗∗∗ represents *p* ≤ 0.001; *N* = 4, *n* = 12, a total of 120 cells in each group analyzed). (C) Correlation between the epigenetic markers and the nuclear circularity on different microgrooved substrates on day 2.

Biophysical alterations to cell and nuclear shape and cellular stiffness suggested underlying modulations to biochemistry, including the epigenetic landscape. To check for epigenetic changes directly, we assessed the extent of the key neuronal epigenetic markers of acetylation of histone H3 at K9 and K14 (AcH3) and histone H4 at K5, K8, K12, and K16 (AcH4), and histone H3 (trimethyl K9) (H3K9me3)^15^ in hNSCs on substrates presenting varying topography via immunofluorescent imaging. Images suggested an accumulation of these markers with increasing topography (Figure 4A). Densitometric analysis of 120 single cells across four independent experiments illuminated quantitative differences for all markers, consistent with the representative images (Figure 4B). Imaging data clearly indicate that hNSCs display epigenetic alterations when cultured on substrates presenting microgrooves.

Whether there may be a link between epigenetic alterations and the nuclear shape change we observed previously (Figure 2) remained unclear. To test this, we plotted epigenetic biomarkers versus nuclear circularity and observed a trend that indicated that more circular nuclei (which were observed on flat, control substrates) also had lower levels of epigenetic biomarkers than more elongated nuclei (which were observed on substrates presenting microgrooves) (Figure 4C). Collectively, our data show that the biophysical changes to hNSCs on substrates presenting microgrooves elicit epigenetic alterations on the cultured cells.

### 3.5 Microgroove Topography Modifies Dynamics of Cell Proliferation, Differentiation, and Neural Rosette Formation

**Figure 5:**
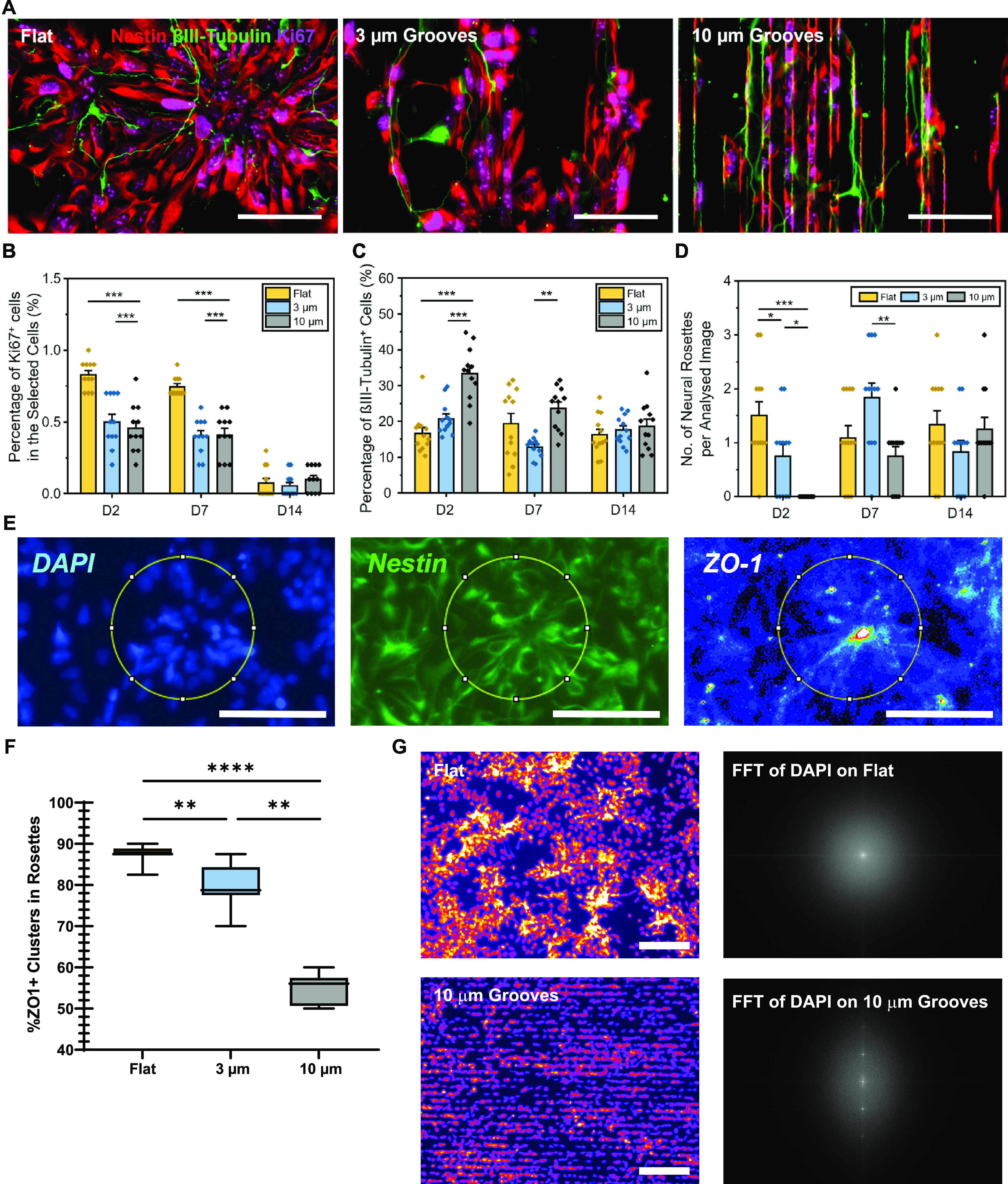
Effects of microgrooved topography on cell proliferation, differentiation, and neural rosette formation in hNSCs. (A) The neural rosette-like structure on flat PDMS (left), 3 μm grooves (middle), and 10 μm grooves (right) grooves on day 2 (nestin, red; β3-tubulin, green; Ki67, magenta; scale bars = 50 μm). (B) Cell proliferation, (C) neuronal differentiation, and (D) number of neural rosette-like structures on days 2, 7, and 14 on different microgrooved substrates (one-way ANOVA with post hoc Tukey’s test was used; the results represent means ± s.e.m.; ∗ represents *p* < 0.05; ∗∗ represents *p* ≤ 0.01; ∗∗∗ represents *p* ≤ 0.001; *N* = 4, *n* = 11–12). (E, F) Neural rosette formation was examined on different substrates by immunostaining of DAPI (blue), nestin (green), and a neural rosette-specific marker, ZO-1 (LUT pseudocolour map applied) (circles: created ROIs based on the average rosette size of 120 μm; scale bars = 100 μm) and the percentage of the ZO-1 clusters in the rosette-like structures was analyzed (one-way ANOVA with post hoc Tukey’s test was used; the results represent median ± IQR; ∗∗ represents *p* ≤ 0.01; ∗∗∗∗ represents *p* ≤ 0.0001; *n* = 7–8). (G) Nuclear orientation of cells and fast Fourier transform (FFT) image analysis for nuclear alignment, showing representative images from each culture (left) and an average FFT graph calculated based on 8–9 randomly selected fields for each topography (right).

While the coordinate modulation of biophysical changes (cell shape, nuclear shape, cell topography, and cell stiffness) and epigenetic biomarkers is clear, the functional impact of substrates presenting microgroove topography remained unknown. To address this, we cultured hNSCs on microgroove substrates and measured proliferation via Ki67^+^, differentiation via βIII-Tubulin^+^, and neural rosette formation over time (Figure 5A–D).

Proliferation at day 2 and day 7 was significantly down-regulated on 3 μm depth grooves (day 2: 50 ± 5%; day 7: 40 ± 4%) and 10 μm depth grooves (day 2: 45 ± 5%; day 7: 41 ± 5%) compared to the flat PDMS (day 2: 83 ± 3%; day 7: 75 ± 2%). By day 14, the percentage of proliferating cells was <10% on, and not statistically different between, all substrates (Flat: 7 ± 3%; 3 μm depth: 5 ± 2%; 10 μm depth: 10 ± 3%) (Figure 5B). These data demonstrate that the microgrooves affect cell cycle progression and suggest that they may in fact accelerate neuronal differentiation.

To assess how topology impacts neuronal differentiation directly, we measured the percentage of βIII-tubulin^+^ cells over time. At day 2 and day 7, the 10 μm depth grooves exhibited the most neuronal differentiation (day 2: 33 ± 2% and Figure S6; day 7: 24 ± 2%). However, at day 14, all the substrates exhibited similar neuronal differentiation, and there was no significant difference between the percentage of neuronal cells (Flat: 16 ± 2%; 3 μm depth: 18 ± 1%; 10 μm depth: 19 ± 2%) (Figure 5C). These data indicate that 10 μm deep microgrooves increase differentiation at early (days 2 and 7), but at the latest time point (day 14) topology has little effect.

A characteristic feature during hPSC neural development is neural rosette formation. Neural rosettes are composed of radially organized columnar neuroepithelial cells and resemble the neural tube structure *in vivo*.^29^ The ability of microgrooves to modulate proliferation and differentiation suggested that they might also impact the formation of neural rosettes. We therefore measured neural rosette formation over time. At day 2, the flat surface clearly retained the neural rosette-like structures while the 3 μm depth grooves rearranged cell alignment along concave microgrooves, moderately affecting the rosette formation (Figure 5A). A severe disruption of rosette formation was observed on the 10 μm depth grooves, where cells and nuclei appeared highly aligned to and fitted mostly within, the microgrooves such that the radially arranged rosette-like organizations disappeared. When we analyzed day 2 images, we found 1.50 ± 0.26 rosettes per image on flat substrates, 0.75 ± 0.22 rosettes on 3 μm deep microgrooves, and no neural rosette formation on 10 μm deep microgrooves (Figure 5D). By days 7 and 14, the 10 μm depth microgrooves no longer had statistically different number of rosettes per image as the flat control substrate. To further assess neural rosette formation, we assessed clustering of the neural rosette-specific marker, ZO-1, within presumptive neural rosettes on day 2 in hNSCs cultured at the same density on different substrates. Quantitative image analysis revealed that hNSCs growing on microgrooved substrates had a decreased number of ZO-1 clusters that are associated with forming rosettes and that this failure to form rosettes is most prominent on the 10 μm deep microgrooves (Figure 5E,F).

Because the loss of rosette structures is likely a key cause of the observed increase in neuronal differentiation observed via βIII-tubulin^+^ data in Figure 5C (hNSCs are not capable of efficient self-renewal outside rosettes), we reasoned that topography might be preventing the formation of the typical radial pattern of cells in rosettes. This was supported by data showing cells on microgrooves are highly oriented (Figures 1C–E and 2A,B). To further interrogate the spatial patterning of cell nuclei imposed by the microgrooves, we performed fast Fourier transform (FFT) analysis of cell nuclei on flat control and 10 μm depth microgrooves. The FFT analysis clearly revealed that cell nuclei are preferentially arranged in a repeating pattern within the microgrooves, while cells on flat surfaces are free to reorient to form rosettes (Figure 5G). Collectively, these data reveal that although microgrooves enhance neuronal differentiation, they significantly diminish the capacity of cells to form neural rosette structures.

### 3.6 Microgroove Topography Modulates Notch Signaling of hNSCs

**Figure 6:**
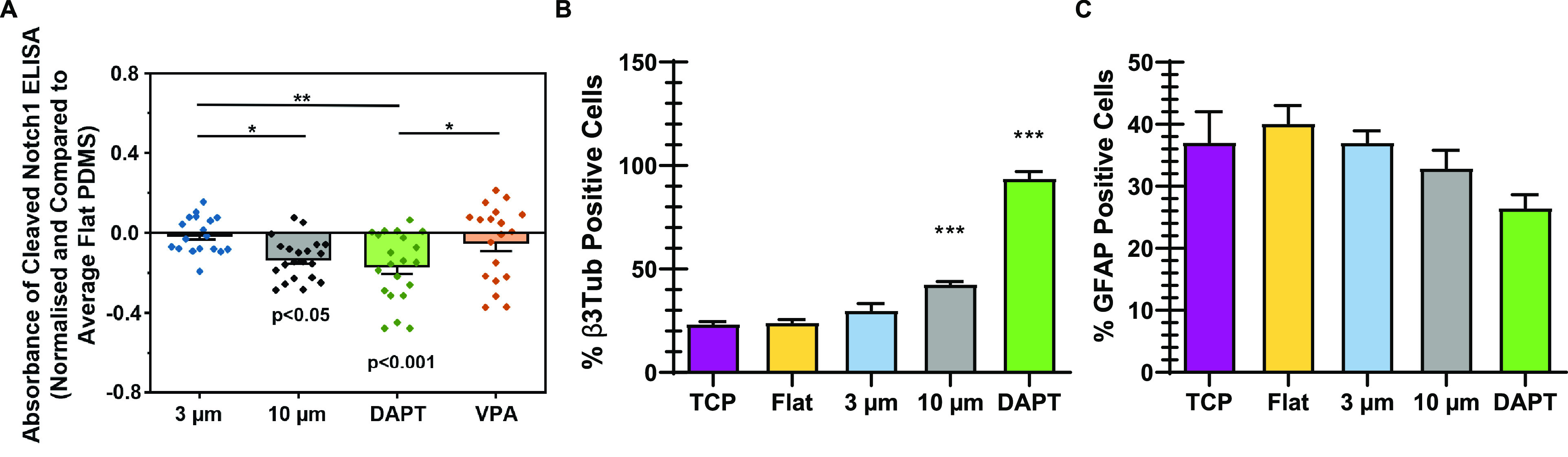
Microgrooves modulated Notch signaling of hNSCs. (A) Effects of microgrooves, DAPT, and VPA on Notch signaling in hNSCs on day 2 were examined by using the cleaved Notch 1 ELISA. The absorbance of the ELISA assay was normalized and compared to the flat PDMS (one-way ANOVA with post hoc Tukey’s test was used; the results represent means ± s.e.m.; ∗ represents *p* < 0.05; ∗∗ represents *p* ≤ 0.01; written *p* values in the figure represent the *p* values compared to the flat PDMS; *N* = 4, *n* = 18–21). Neuronal (B) and astrocytic (C) differentiation on different microgrooved substrates at day 7 in culture, comparing the effect of different topographies to the administration of DAPT (one-way ANOVA with post hoc Dunnett’s test was used; ∗∗∗ represents *p* ≤ 0.001 compared to flat PDMS; *N* = 3; *n* = 15).

Notch signaling has previously been implicated in many neural systems^46^ but is typically dependent on cell–cell contacts. We therefore asked how microgroove topography—which modulates cell–cell contacts—regulates Notch signaling in our system. To address this, we measured cleaved Notch1 via an ELISA assay as a measure of Notch signaling on flat, 3 μm deep microgrooves, and 10 μm deep microgrooves. We found that the 10 μm deep microgrooves significantly downregulated cleaved Notch1 relative to both 3 μm deep microgrooves and flat controls (Figure 6A). Moreover, a Notch signaling inhibitor, *N*-[*N*-(3,5-difluorophenacetyl)-l-alanyl]-*S*-phenylglycine *tert*-butyl ester (DAPT), downregulated cleaved Notch1 levels to a similar extent as the 10 μm deep microgrooves. Finally, the histone deacetylase inhibitor valproic acid (VPA), did not downregulate cleaved Notch1, indicating that epigenetic modulations do not regulate Notch1 signaling. Thus, our prior results (Figure 4) indicated that microgrooves impact epigenetic modifications, and the results here (Figure 6A) indicate that 10 μm deep microgrooves impact Notch signaling. Taken together, our data indicate that topographic features regulate Notch1 signaling and epigenetics in tandem.

As a further control, we compared the effect of Notch signaling inhibition and topography on the differentiation potential of neural stem cells and astroglial progenitors obtained from the same cell source with a glial conversion protocol previously described.^47^ While neuronal progenitors are highly sensitive to Notch signaling inhibition and respond to it by readily differentiating, the same pathway has less direct effects on the differentiation of glial progenitors. Indeed, we observed that both DAPT based Notch signaling inhibition and the increasing depth of the microgrooves correlated to increased differentiation of neuronal progenitors, while it had no significant effect in the astrocyte progenitors (Figure 6B,C). This further indicates that topography is partially phenocopying Notch signaling inhibition in neuronal cells, and we propose this might be achieved by restricting cellular movement and reducing their capacity to forming rosettes.

## 4 Discussion

To date, neuronal behaviors from immortalized neuronal cell lines, primary neurons isolated from both animal CNS and PNS, and neurons derived from human and animal PSCs have been studied with various topographical models. Despite these efforts, the cellular mechanisms underlying detection and responses to topographic cues remain elusive.^48^ In this study, we demonstrated topographical control of cell fate in human clinically relevant neural cells derived from hPSCs and illuminated changes to the epigenetic landscape and modulation of Notch signaling via microgroove topology.

We found that seeding hPSC-derived NSCs on substrates with 10 μm groove/ridge width led to increased alignment on the 3 and 10 μm depth microgrooves, similar to previous studies using other cell types and various nano-/microgrooved patterns.^49−60^ While neural cells were shown to discriminate depth of the nano-/microgratings in previous studies, the hPSC-derived NSCs on the 10 μm depth grooves also exhibited a higher degree of alignment compared to the 3 μm depth grooves. Prior literature suggested that topographical patterns with deeper grooves may serve as physical guidance for neurite extension. The ridges with higher steps act as barriers, where cytoskeletons were too stiff to bend across. Consistently, we found via AFM that cell stiffness was higher on the microgrooves compared to flat, control substrates. While cell stiffness was higher on the 3 μm depth grooves compared to the flat substrates, a statistical significance was only found on the 10 μm depth grooves compared to the flat control. Previously, research has shown that cell spreading and polarization may be caused by two different mechanisms, including space constraint and adhesion induction.^61^ Nanogrooved substrates induce cellular polarization via focal adhesion formation and enhancement of intracellular traction forces via RhoA/ROCK activation.^61^ On the other hand, cells on microgrooved substrates, which trigger cellular polarization via spatial constraint, acquire almost no visible focal adhesions but only directionless pseudopodia-based adhesion. The increased cell stiffness observed on our microgrooved platforms via AFM is proposed to be a reflection of cell tension unrelated to focal adhesion-exerted intracellular traction forces. Previously, it was shown that early contact guidance in grooved substrates only requires simple cellular machinery, such as actin polymerization.^62^ Cells on the 3 μm depth grooves are not fully docked within the grooves, allowing actin polymerization with a less specific direction compared to the 10 μm depth grooves. On the other hand, actin fibers of cells constrained within the 10 μm depth grooves polymerize toward the contact surfaces, resulting in an enhancement in cell–substrate alignment, cell elongation, and thus cell tension. Similar to the AFM results, SICM also revealed topographical changes in cells on microgrooves compared to flat substrates, where cells were more deformed on the flat surface compared to the microgrooved substrates. SICM is an emerging imaging technique that not only enables measurement of cellular mechanical properties but also has a high potential for live cell imaging.^63^ Compared to conventional AFM, which acquires cell mechanical mapping through tapping mode, SICM applies no force, thus representing an attractive, nondestructive alternative with a comparable resolution to AFM.^64^

Previously, substrate-based biophysical cues, incorporating either topographic patterns or anisotropic mechanical strain, have been reported to modulate the epigenetic status of cells. Although there have been a few studies on topographical epigenetic modulations recently,^12,13,32,65−67^ limited research has focused on neuronal systems to date. Furthermore, because we use human clinically relevant cell systems, our study may reveal distinct biophysical regulatory mechanisms contributing to human neural development. The most significant effects were shown at day 2, where epigenetic markers of AcH3, AcH4, and H3K9me3 in hPSC-derived NSCs were enhanced on microgrooves, and increasing groove depth elicited higher expression compared to the flat substrates with a higher degree of statistical significance on the 10 μm depth grooves compared to the 3 μm depth grooves. The upregulation of the epigenetic markers also correlated to nuclear elongation found on the microgrooved substrates. Microgrooves have been shown to increase AcH3 and H3K4 methylation in adult fibroblasts, in turn promoting a mesenchymal-to-epithelial transition, further increasing their reprogramming efficiency into iPSCs.^13^ An increase in AcH3 in MSCs was also reported on the 3 μm depth grooves with 10 μm ridge/groove width.^12^ Overall, while upregulations of AcH4 and H3K9me3 were more significant, AcH3 is partially increased in hPSC-derived NSCs. Previous studies have shown the correlation between AcH3 and AcH4 and multiple neuronal growth genes, such as NeuroD and BDNF; AcH3 and AcH4 were found upregulated in neuronal extracts compared to undifferentiated extracts.^68,69^ Because the alterations of these epigenetic markers play a key role in neuronal development, upregulation of AcH3 and AcH4 points to promotion of neuronal differentiation. Methylation of H3K9, a repressive marker associated with gene silencing and heterochromatin formation, has been recognized as a crucial regulator during neurogenesis. While H3K9me and H3K9me2 are related to reversible gene repressions, the H3K9me3 maker is responsible for long-term gene repression.^70^ Our results showed that the microgrooved topography could enhance H3K9me3 in hPSC-derived NSCs. Previously, others showed that microgrooved topography can enhance the expression of a subunit of H3 methyltransferase (WDR5) and result in an increase in H3K4me3 in fibroblasts.^13,67^ Our recent work with immunogold labeling of H3K9me3 in hiPSC-derived NSCs demonstrated that the number of H3K9me3 foci (normalized to the nuclear volume of the cell) in the cells on the 10 μm grooves were significantly higher than the cells on the flat surface, which is consistent with results using immunofluorescent staining in this study.^71^ Furthermore, the H3K9me3 foci were predominantly distributed in the peripheral nuclear region, which might further contribute to formation of heterochromatin, thus silencing gene expression and lead to progression of neuronal differentiation.^15^

Significant changes to nuclear shape in response to microgrooved substrates and the correlation of nuclear shape with epigenetic modification were found by us and several others.^12,13,32,72,73^ Possible mechanisms have been proposed, including (1) the microgroove-induced nuclear elongation could affect nuclear pores and the spatial distribution of epigenetic modulators via nucleocytoplasmic shuttling^12,74^ and (2) the mechanical stress exerted by microgrooved-induced alignment could affect the nuclear membrane and nuclear matrix and therefore regulate cellular responses through mechanotransduction.^75^ Herein, we applied the state-of-the-art FIB-SEM 3D tomography to slice, reconstruct, and analyze nuclei at high spatial resolution to assess changes on the microgrooves. This revealed that nuclei of cells on microgrooves exhibited intricate 3D morphology and in particular significantly more nuclear invaginations. Moreover, our FIB-SEM findings were consistent with confocal images of Lamin A/C, lending further support for the existence of nuclear invaginations. We propose that the invaginations facilitate nucleocytoplasmic transport and signaling, chromatin remodeling, and potentially calcium signaling, as suggested previously.^76^

Although an abundance of research has reported topographical effects on cell alignment and neurite guidance, only recently the influence of topography on cell differentiation has been revealed.^2,8,9,77−81^ Similar to previous findings, our results showed that hPSC-derived NSCs decreased proliferative potential and increased neuronal differentiation on the microgrooves at day 2 and day 7. Furthermore, neural rosette formation, a characteristic feature during hPSC neural development, significantly reduced on microgrooved substrates and reduced further as the depth of the microgrooves increased from 3 to 10 μm. As the formation of neural rosettes is highly dependent on cell–cell contacts and multicellular structural self-organization, the microgrooved patterns might act as geometric barriers (deeper microgrooves as a result of greater physical barriers), antagonizing or even preventing rosette formation. Because rosettes are highly dependent on cell–cell contacts, we interrogated the Notch signaling pathway. Indeed, microgrooves inhibited Notch1 signaling similar to the chemical Notch signaling inhibitor, DAPT. Furthermore, VPA, the epigenetic inhibitor, did not alter Notch signaling.

Previously, HDAC/silencing mediator of retinoid and thyroid hormone receptors (SMRT) complexes have been shown to inhibit the Notch signaling pathway by repressing the transcription of its downstream genes, such as Hes1.^82,83^ HDACs may also regulate neurogenesis through changes in Notch target gene expression by histone deacetylation.^84^ Furthermore, the HDAC inhibitor, VPA, leads to an activation of the Notch signaling cascade by inducing a γ-secretase dependent activation of Notch signaling, resulting in increased levels of NICD.^85^ In our studies, there was a trend of enhanced histone acetylation while Notch signaling was significantly downregulated on the microgrooved substrates. Although previous studies have shown that Notch is downstream of HDAC signaling, multiple mechanisms have been involved for different target genes at different stages.^82−85^ Future research could explore a more detailed mechanism of their regulatory machinery, including examination of the expression of HDACs and histone acetyltransferases, as well as HDAC activity. Chromatin immunoprecipitation sequencing and bisulfite sequencing could be used to identify the downstream genes affected by the observed epigenetic regulations. A cell line harboring a Notch reporter can be further utilized to study the temporal changes on Notch signaling correlated to the modifications of epigenetic state and further decipher the interactions between the two mechanisms.

Our study demonstrates that Notch signaling can be modulated by topographical cues, simply via geometrical constraints provided by the microgrooved platform. Previously, modulation of Notch signaling has been achieved with bioengineering systems based on biomaterials or cells, including tissue-culture polystyrene plates immobilized with ligands,^86^ coculture of cells expressing Notch receptors and Notch ligands,^87^ or cells transfected with active Notch intracellular domains (NICD).^88^ Dynamics of Notch signaling have been investigated by quantitative time-lapse live imaging of cells harboring a Notch activity reporter in the presence of surface-immobilized Notch ligand and in culture with synthetic cells harboring a Notch ligand.^89^ To mimic cell–cell interactions, recent studies have further immobilized cell-surface ligands, such as Notch and Jagged1 ligands, to different biomaterial surfaces.^88,90−92^ Despite previous methods showing successful modulation of Notch signaling, cell coculture and transfection of NICD have limitations including cell heterogeneity, confounding effects from diverse, complex signaling pathways within different cell types, and low transfection efficiency. There are also drawbacks to the previously described material-based approach as it is challenging to control ligand orientation and their accessibility. Furthermore, these platforms generally require surface modification schemes, where complicated treatment process and advanced surface characterization are often necessary. In contrast, we here use biophysical and topographical cues to modulate Notch signaling. The microgrooved platform may present a facile and powerful method to control cell signaling, and with optimized parameters, it can be applied to facilitate stem cell maintenance or to regulate cell fate decisions.

Recently, in addition to structural support, biophysical considerations in the rational design of biomaterials and biointerfaces have been shown to direct cell guidance, cell signaling, and cell fate.^93,94^ Similar to our study, previous reports have presented promising results in controlling cellular responses and cell fate through topography instead of conventional biochemical induction.^95^ These designs based on cell–substrate interactions can be integrated into implantable biomedical devices and microfluidics devices, to fine-tune the control of cell proliferation, differentiation, tissue integration, and thus the overall performance.^95,96^ The results in this study elucidate topographical modulations of cell epigenetics and Notch signaling based on microgrooved platforms. These mechanisms can be applied to the material or interface design to generate optimal microenvironments for a target cell type or tissue for clinical applications and *in vitro* studies, which required enhanced, long-term cell culture.

## 5 Conclusion

We reported that our microgrooved platform can enhance cell alignment, change nuclear shape, and increase cell stiffness. It can also regulate epigenetic status of cells as well as cell proliferation and differentiation. Changes to the epigenetic landscape correlated to changes in nuclear shape. Finally, we found that microgrooves can modulate Notch signaling in parallel to epigenetic modulations. The biophysical regulation of epigenetic effects and Notch signaling could shed light on rational design of new biomaterial interfaces to mimic cell niches tailored for various biological and translational applications.

## Supporting Information Available

Additional experimental details and results, including protein expression of epigenetic markers of hESC-derived and hiPSC-derived NSCs on different substrates at day 2 in Figure S1, fabrication and patterns of different PDMS substrates used in the study in Figure S2, Young’s modulus of the fabricated PDMS substrates measured by AFM in Figure S3, examples of cells examined by using FIB-SEM in Figure S4, alignment between nuclear polarization and orientation of nuclear invagination in Figure S5, and the results of real-time quantitative reverse transcription polymerase chain reaction (qRT-PCR) on neuronal differentiation at day 2 in Figure S6 (PDF)

## Notes

Research raw data are available upon reasonable request from rdm-enquiries@imperial.ac.uk.
