# Supplementary material for: Biophysical Regulations of Epigenetic State and Notch Signaling in Neural Development Using Microgroove Substrates
Source: ACS Appl Mater Interfaces. 2022 Jul 13;14(29):32773–87. doi: 10.1021/acsami.2c01996 (PMC9335410; doi:10.1021/acsami.2c01996)
Supplement: Supplementary file 1 — am2c01996_si_001.pdf [file am2c01996_si_001.pdf]

Supporting Information

Biophysical Regulations of Epigenetic State and Notch Signalling in Neural Development using Microgroove Substrates

Chia-Chen Hsu[a,b,c1], *Andrea Serio*[a,b,c†1], *Sahana Gopal*[a,b,c], *Amy Gelmi*[a,b,c‡], *Ciro Chiappini*[a,b,c†], *Ravi A. Desai*[a,b,c], *Molly M. Stevens*[a,b,c*]

[a] Department of Materials, Imperial College London, UK

[b] Department of Bioengineering, Imperial College London, UK

[c] Institute of Biomedical Engineering, Imperial College London, UK

[†]Current Address: Centre for Craniofacial and Regenerative Biology, King's College London, London, SE1 9RT, UK

[‡]Current Address: School of Science, STEM College, RMIT University, Melbourne, VIC 3001, Australia

[1]These authors contributed equally to this work.

[*]Corresponding author. Department of Materials, Imperial College London, Exhibition Road, London SW7 2AZ. E-mail address: m.stevens@imperial.ac.uk.

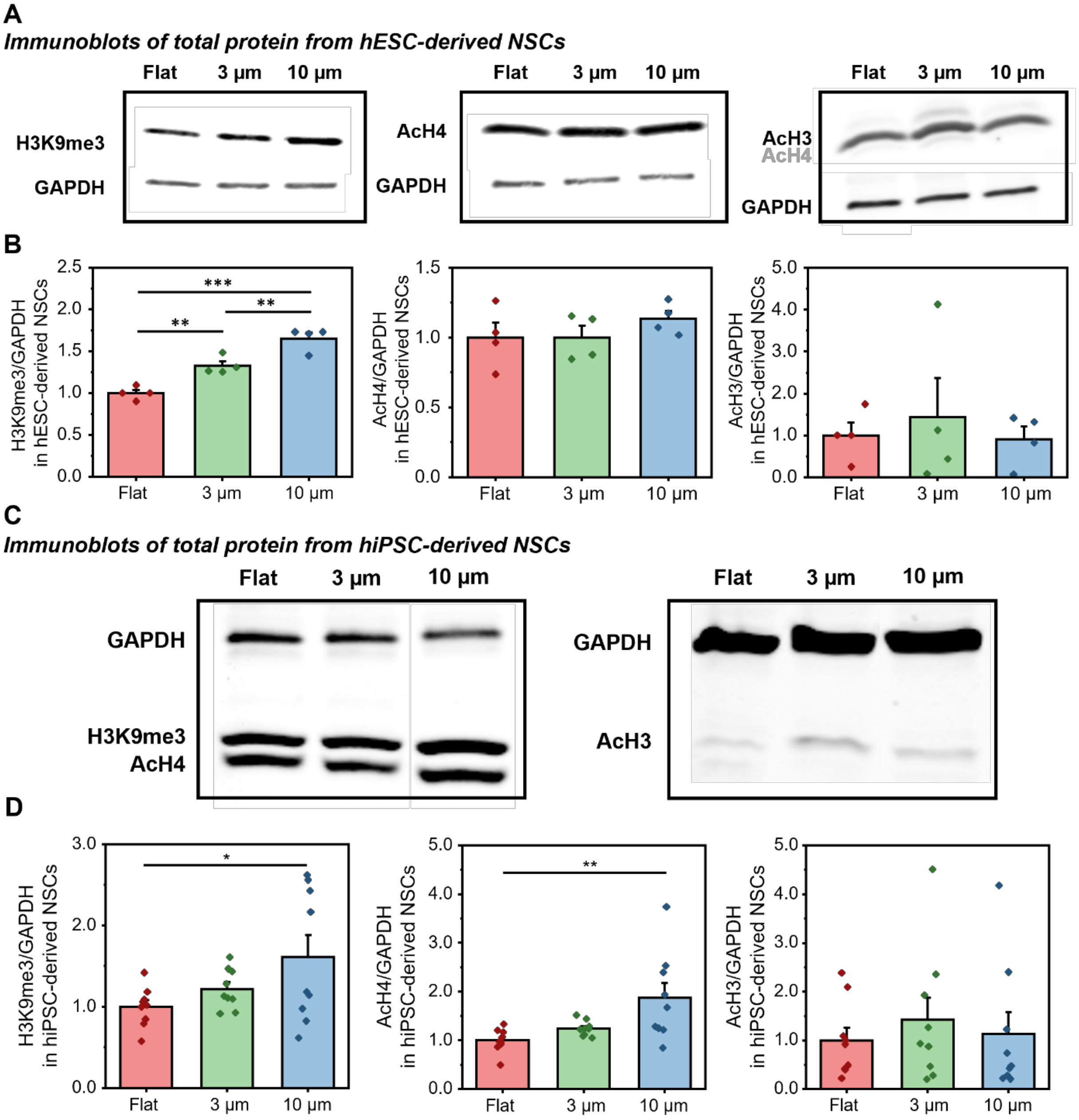

Figure S1. Expression of epigenetic markers of hESC-derived and hiPSC-derived NSCs on different PDMS substrates at Day 2 determined by immunoblotting using the respective antibodies. (A, B) Expression of epigenetic markers, including H3K9me3, AcH4, and AcH3 in hESC-derived NSCs on different PDMS substrates at Day 2. (C, D) Expression of epigenetic markers, including H3K9me3, AcH4, and AcH3 in hiPSC-derived NSCs on different PDMS substrates at Day 2. (Flat

represents the flat PDMS; 3 μm and 10 μm represent 3 μm-depth and 10 μm-depth grooves respectively; one-way ANOVA with post hoc Tukey's test was used; the results represent means ± s.e.m. * represents $p < 0.05$; ** represents $p \leq 0.01$; *** represents $p \leq 0.001$; for hESC-derived NSCs, N = 4, n = 4; for hiPSC-derived NSCs, N = 3, n = 9.)

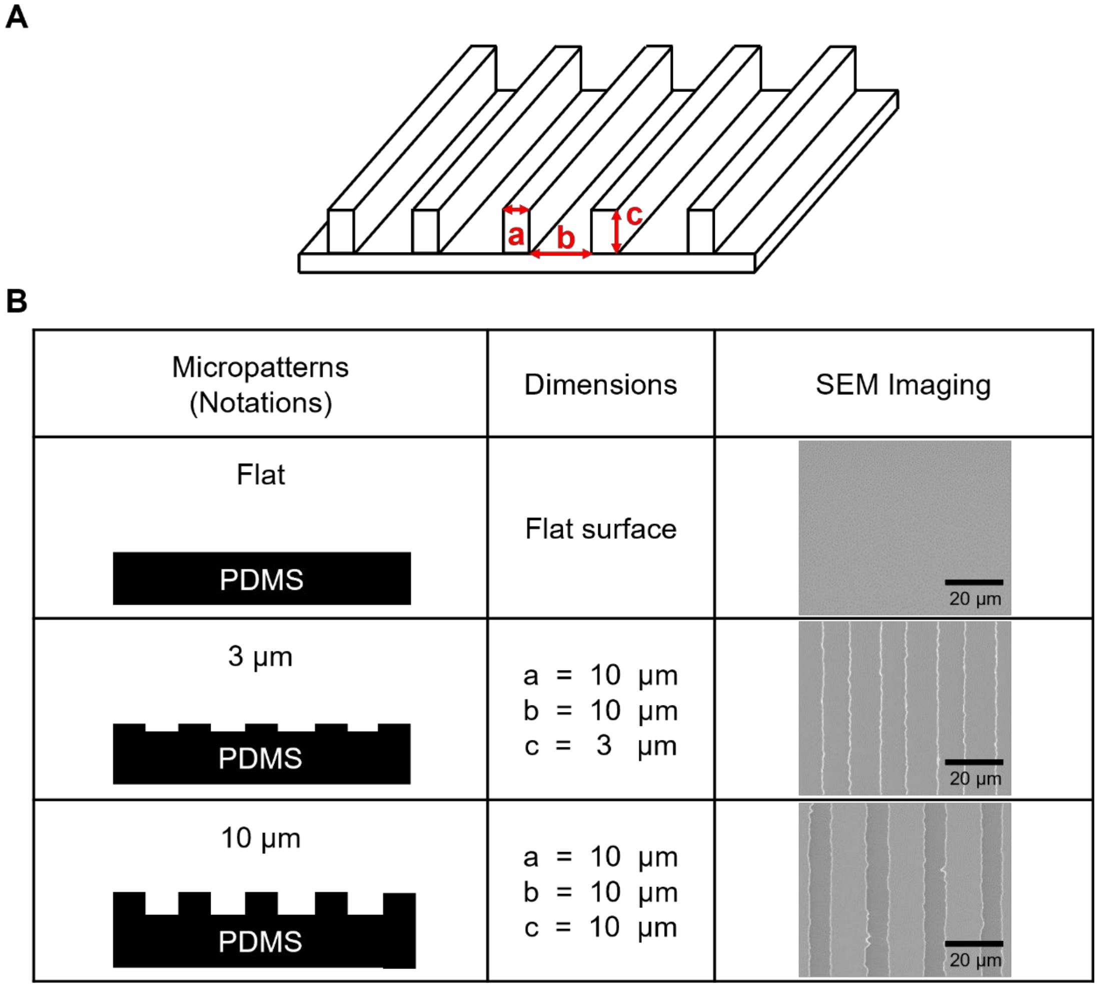

Figure S2. Fabrication and patterns of different PDMS substrates used in the study. (A) Three-dimensional (3D) schematic of the microgrooves, where **a** is the width of the ridge, **b** is the width of the groove, and **c** is the depth of the groove. (B) Patterns of the flat PDMS and the microgrooves used in the study with their notations, dimensions presented as **a**, **b**, **c** in (A), and their scanning electron microscopy (SEM) images (scale bars = 20 μm).

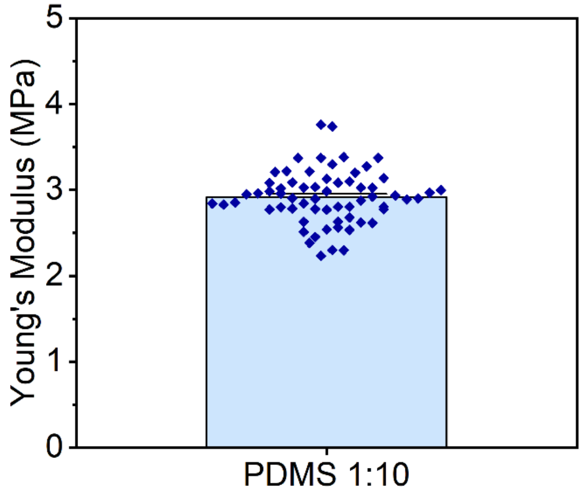

Figure S3. The Young's Modulus of the fabricated PDMS substrates measured by AFM (N = 2, n = 64).

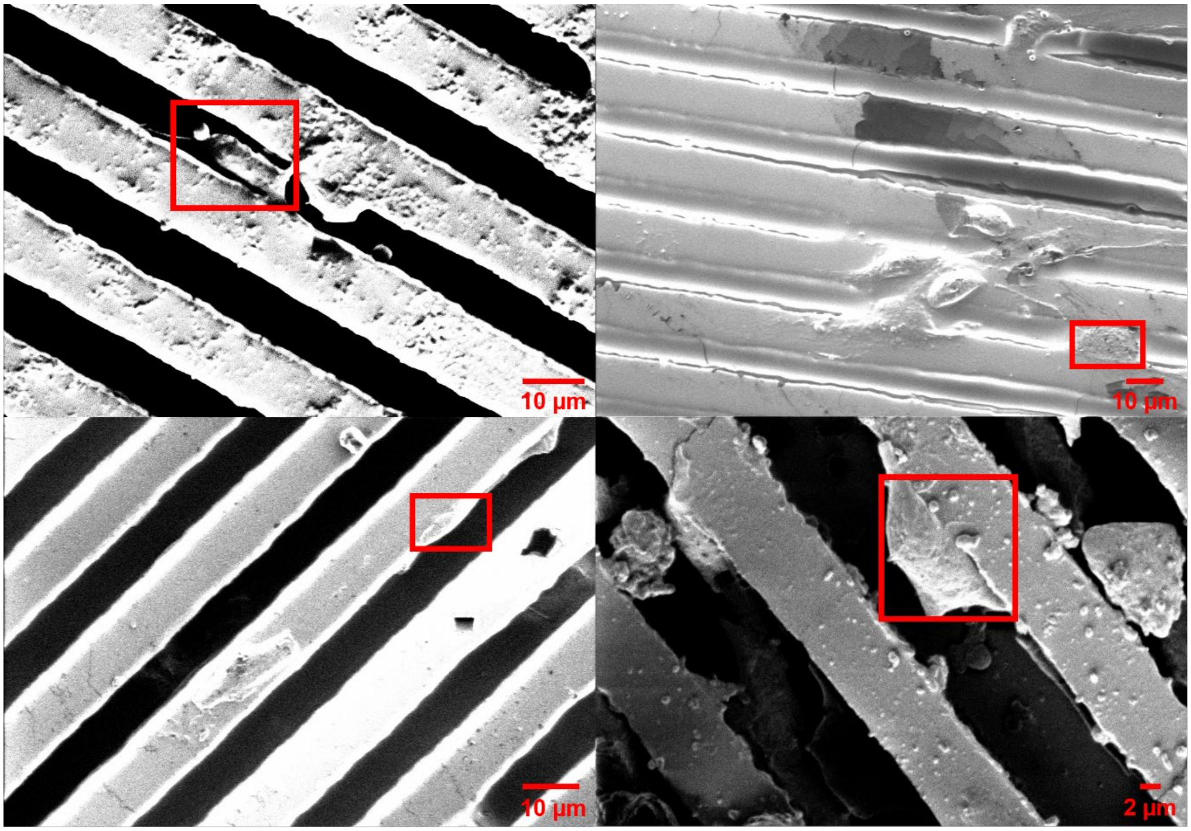

Figure S4. Examples of cells examined using FIB-SEM. Cells docked within 10 μm-depth grooves were specifically selected (red boxes) to examine the cellular effects caused by spatial limitation.

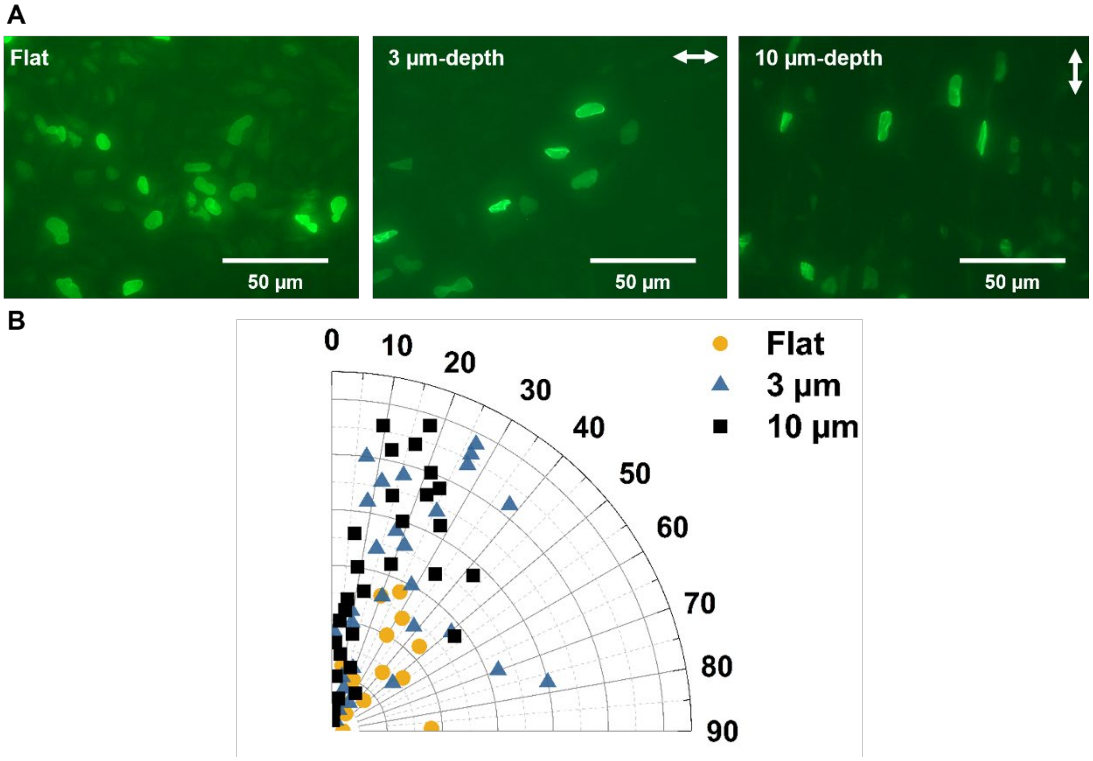

Figure S5. Alignment between nuclear polarisation and orientation of nuclear invagination. (A) Nuclear Lamin A/C staining of hNSCs on flat and microgrooved substrates (white arrows: direction of grooves; scale bars = 50 μm). (B) Polar graph showing the angles between nuclear polarisation (defined by the Feret's diameter of a cell nucleus) and the orientation of nuclear invagination.

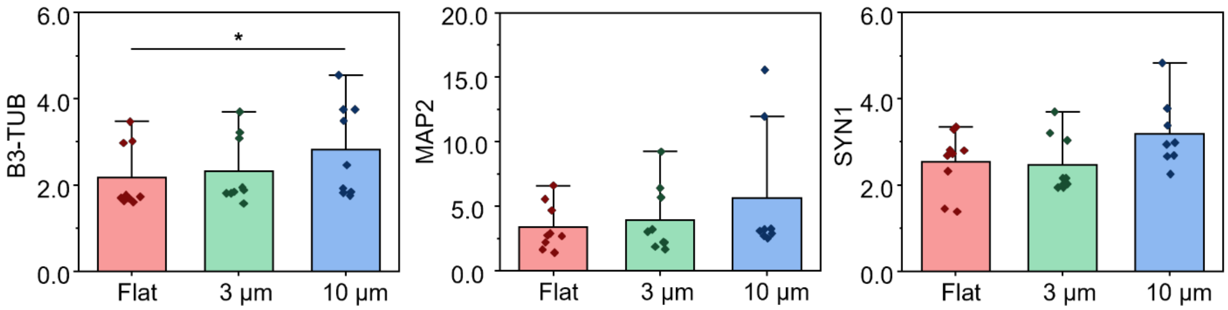

Figure S6. Results of quantitative real-time reverse transcription polymerase chain reaction (qRT-PCR) on neuronal differentiation at Day 2, where microgrooves showed an increased trend of the neuronal differentiation markers, including neuron-specific cytoskeletal markers, βIII-tubulin and MAP2, and SYN1, a synaptic marker indicating neuronal development, density, and integration. As the nucleic acids were collected from the whole membranes (on both the ridge and microgroove regions), the effects were less significant compared to immunofluorescence imaging analysis. (One-way ANOVA with post hoc Dunnett's test was used; * represents $p < 0.05$; hiPSC-derived NSCs, N = 3, n = 8-9.)

Materials and Methods

Protein Extraction and Immunoblotting for the Selected Epigenetic Markers

Cells on different substrates were lysed with radioimmunoprecipitation assay (RIPA) lysis buffer supplemented with phenylmethylsulfonyl fluoride in dimethyl sulfoxide (DMSO), a protease inhibitor cocktail in DMSO, and sodium orthovanadate in water (Santa Cruz Biotechnology, USA) on ice. The collected protein lysates were centrifuged and the protein in the supernatant was quantified with the DC Protein Assay (Bio-Rad, UK) and stored at -80 °C. To perform an immunoblotting, the protein samples were run in SDS/PAGE with a Mini-PROTEAN® System (Bio-Rad) and transferred to PVDF membranes with a Trans-Blot® Turbo™ Blotting System (Bio-Rad). Membranes were blocked with 5 % (w/v) bovine serum albumin (Sigma-Aldrich) in Tris-buffered saline containing 20 mM Tris and 100 mM NaCl (pH 7.6), followed by incubation with the primary antibodies, including histone H3 (acetyl K9 + K14) (1:1000; Cell Signaling Technology, USA), histone H4 (acetyl K5 + K8 + K12 + K16) (1:1000; Abcam, UK), histone H3 (trimethyl K9) (1:1000; Abcam, UK), and GAPDH (1:1000; Santa Cruz Biotechnology) diluted in

Tris-Buffered Saline Tween-20 (TBST) buffer containing 20 mM Tris, 100 mM NaCl, and 0.1 % (v/v) Tween-20 at 4 °C overnight. After 3 washes with TBST, infrared secondary antibodies (1:1000; LI-COR, USA) were then incubated at room temperature for 1 h. After 3 washes with TBST, the imaging and data analysis were performed with an Odyssey Imaging system (LI-COR, USA).

Image Analysis of Alignment Between Nuclear Polarisation and Orientation of Nuclear Invagination

The fluorescent images of hPSC-derived NSCs stained with Lamin A/C were analysed by measuring the angle between nuclear polarisation (defined by the Feret's diameter[1] of a cell nucleus) and the orientation of the nuclear invagination using the Angle Measurement Tool of ImageJ 64 (Version 2; NIH). 6 images per experimental group, 5 cells (maximum) per selected field, and a total of 30 cells (maximum) in each group were analysed. The result was plotted as a polar graph using Polar r(X) theta(Y) of OriginLab.

RNA Isolation and qRT-PCR

Cells were lysed using TRIzol® (Thermo Fisher Scientific, UK) and the RNA was subsequently extracted and purified using the Direct-zol™ RNA MiniPrep Kits (Zymo Research, USA) according to the manufacturer’s instructions. RNA concentration and purity were determined with a Nanodrop spectrophotometer (Thermo Fisher Scientific). cDNA was then reverse transcribed from the RNA using a QuantiTect® Reverse Transcription Kit (Qiagen, UK). cDNA samples with low concentrations, resulted from the low efficiency during RNA extraction presumably due to the interference of the underlying substrates. cDNA samples were pre-amplified without introducing bias with a TaqMan™ PreAmp Master Mix Kit (Thermo Fisher Scientific). TaqMan™ qRT-PCR

assays were performed and analysed using a QuantStudio^{TM} 6 Flex Real-Time PCR System (Applied Biosystems, USA) with annealing and extension at 60 °C and denaturing at 90 °C. The following table is a list of the PCR primers used and these primers were synthesised by Life Technologies (Table S1).

Table S1. Primers used in this study.

Target Gene	Assay ID
GAPDH	Hs02758991_g1
TUBB3	Hs00801390_s1
MAP2	Hs00258900_m1
SYN1	Hs00199577_m1

Reference

1. Walton, W. H., Feret's Statistical Diameter as a Measure of Particle Size. *Nature* **1948,** *162* (4113), 329-330.
